# Genome-Wide Identification and Expression Analysis of the bHLH Transcription Factor Family and Its Response to Abiotic Stress in Mongolian Oak (*Quercus mongolica*)

**DOI:** 10.3390/cimb45020075

**Published:** 2023-01-31

**Authors:** Hao Zhan, Hanzhang Liu, Wanfeng Ai, Xiaoyi Han, Yu Wang, Xiujun Lu

**Affiliations:** 1 College of Horticulture, Shenyang Agricultural University, Shenyang 110866, China; 2 College of Forestry, Shenyang Agricultural University, Shenyang 110866, China

**Keywords:** *Quercus mongolica*, *bHLH* gene family, genome-wide analysis, expression pattern, abiotic stress

## Abstract

The basic helix-loop-helix (bHLH) family, one of the largest families of transcription factors in plants, is extensively involved in the growth, development, and stress response of several woody plants. However, no systematic analysis of the bHLH gene family in *Quercus mongolica* has been reported. We characterize *QmbHLH* genes and identify the functions of QmbHLH proteins in *Q. mongolica*. We used bioinformatics approaches, qRT-PCR analysis, and RNA sequencing data to examine chromosomal distributions, gene structures, and conserved patterns, and identified 89 *QmbHLH* genes, which were divided into 21 subgroups based on the phylogenetic analysis of *bHLH* genes in *Arabidopsis thaliana*. Segmental replication played a more prominent role than tandem duplication in the expansion of the *QmbHLH* gene family. Based on patterns of tissue-specific expression, protein interactions, and cis-element analysis, *QmbHLH* genes may be extensively involved in the growth and development of *Q. mongolica*. In leaves, stems, and roots, 12 selected *QmbHLH* genes exhibited responsiveness to abiotic stresses (salt, cold, weak light, and drought). Our study facilitates follow-up functional investigations of the *bHLH* gene family in *Q. mongolica* and provides novel insights into *bHLH* superfamilies in woody plants.

## 1. Introduction

Transcription factors are the most enriched gene modulators in multicellular genomes and repress or activate gene expression by binding to certain DNA sequences, thereby playing unique regulatory roles in many vital biological processes in plants, such as growth, development, stress responses, and signal transduction [1,2]. Basic helix-loop-helix (bHLH) proteins are part of a transcription factor superfamily widely found in fungi, animals, and plants [3]. The bHLH structural domain consists of approximately 60 amino acids, which form an α-helix1–loop–α-helix2 structure (helix-loop-helix; HLH) and a basic region. The basic region is composed of approximately 15–20 amino acids that can bind to DNA. These conserved amino acids recognize the E-box (5’-CANNTG-3’) [2,4]; most bHLHs that bind to the E-box do so by binding to the G-box (5’-CACGTG-3’) [5,6,7]. The structure of the α-helix–loop–α-helix depends on hydrophobic amino acid interactions that facilitate the formation of homodimers or heterodimers [1]. Consequently, bHLH transcription factors frequently function as dimers. HLH proteins lacking basic regions can form dimers with bHLH proteins but have no DNA-binding capacity.

Current research has shown that plant bHLH transcription factors are involved in modulating a variety of assimilation pathways and signal transduction pathways, such as pathways associated with light signal transduction [8,9], secondary metabolism [10,11], organ development [12,13,14,15], and stress response [16,17,18,19]. Under stress conditions, certain bHLH transcription factors bind to the promoters of critical genes in diverse signaling pathways, such as those involved in photosynthesis, hormone metabolism, and enzyme systems. By modulating the transcript levels of these target genes, the transcription factors mediate plant tolerance to stress [20]. For instance, *PebHLH35* is overexpressed in *Populus euphratica* under drought stress. *PebHLH35* improves the drought tolerance of *P*. *euphratica* by regulating stomatal density, stomatal size, and photosynthesis [21]. Furthermore, *bHLH39* in wheat enhances tolerance to salt stress by increasing the expression of stress response genes [22]. *SbbHLH85* in *Sorghum bicolor* [20] and *BvbHLH93* in *Beta vulgaris* [23] also participate in the salt stress response. In addition, *VabHLH1* in *Vitis amurensis* enhances cold stress tolerance by modulating *COR* gene expression [24], while *FtbHLH2* in *Fagopyrum tataricum* [25] and *StbHLH45* in *Solanum tuberosum* [14], as positive regulators of the cold stress response, play a positive role in cold tolerance. *NtbHLH123* is a transcriptional activator in *Nicotiana tabacum* that enhances cold tolerance by modulating ROS scavenging-associated genes and stress response genes [26]. These observations indicate that bHLH transcription factors are highly involved in plant responses to stress.

Oaks are members of the Fagaceae family, which comprises 300 to 600 species of large evergreen and deciduous trees distributed in the temperate and tropical zones, and these trees are an essential part of the forests of the Northern Hemisphere [27]. Mongolian oak (*Quercus mongolica*), which exhibits high levels of resistance to damage, is found in warm, temperate forests. *Q*. *mongolica* is a dominant deciduous species of the genus *Quercus* and is the dominant species in broad-leaved mixed and coniferous forests in northern and northeastern China [28]. *Q*. *mongolica* is among the tree species with the highest comprehensive value. For instance, *Q*. *mongolica* has important functions in greening, soil conservation, carbon sinks, and medicine, and serves as food for an important Chinese silk-secreting insect, the tussah, as the basic biological resource for tussah production. Its acorns, bark, and leaves contain important edible, medicinal, and industrial raw materials. This species is also used for ornamental landscaping, ecological protection, and timber production [28,29].

*Q*. *mongolica* is highly tolerant to drought and cold stress [29]; nevertheless, as a photophilic species, seedlings of *Q*. *mongolica* do not grow properly under conditions of low light. These conditions seriously interfere with the natural regeneration process [30]. Furthermore, few reports have examined whether *Q*. *mongolica* exhibits resistance to other abiotic stresses. Rapid advances in molecular biology could assist in developing an understanding of the molecular mechanisms of abiotic stress responses and facilitate breeding, together with the diversification of *Q*. *mongolica*. In recent years, research on *Q*. *mongolica* has mainly focused on physiology and ecology; however, research on molecular biology, especially the study of gene families, is lacking. We recently sequenced the *Q*. *mongolica* genome [31]. This development provides a better platform for studying the growth and progression of *Q*. *mongolica* at the genome-wide level.

In this study, we thoroughly analyzed the family of *QmbHLH* genes using a variety of bioinformatics approaches and identified the functions of QmbHLHs in the growth and development of *Q*. *mongolica*. Additionally, a combination of qRT-PCR analysis and RNA sequencing data was used to determine the *QmbHLH* expression pattern under abiotic stress. Our findings indicated that *QmbHLH* genes may be extensively involved in the growth, development, and stress responses of *Q. mongolica*. This study lays the groundwork for investigating the biological functions of *QmbHLH*s and the enrichment of abiotic stress–responsive gene pools in woody plants.

## 2. Materials and Methods

### 2.1. Identification of bHLH Genes in Q. mongolica

The genome data of *Q. mongolica* came from our research group (https://www.ncbi.nlm.nih.gov/bioproject/PRJNA609556/, accessed on 2 June 2022) [31]. The Arabidopsis thaliana bHLH protein sequences, downloaded from PlantTFDB (http://planttfdb.gao-lab.org/family.php?fam=bHLH, accessed on 3 June 2022), were compared with the whole protein sequences of *Q. mongolica* using BLASTp by default parameters to obtain the potential *QmbHLH* genes. HMMER software (http://www.hmmer.org/, accessed on 3 June 2022) was used to search for bHLH proteins in the protein sequences of the *Q. mongolica* genome by using the hidden Markov model (HMM) file of the HLH domain (PF00010) downloaded from the Pfam database (https://pfam.xfam.org, accessed on 3 June 2022). The redundant sequences between the two results were removed to obtain the candidate *QmbHLH* gene family members. All presumptive QmbHLH protein sequences were uploaded to CDD-Search (https://www.ncbi.nlm.nih.gov/Structure/cdd/wrpsb.cgi, accessed on 4 June 2022) and SMART (http://smart.embl-heidelberg.de/, accessed on 4 June 2022) databases for manual correction to verify the existence of bHLH domains. In addition, the basic features of QmbHLH proteins, including the length of amino acid sequences (AA), molecular weights (MWs), theoretical isoelectric points (PI), grand averages of hydropathicity (GRAVY), and instability index values (II), were predicted using the ExPASy website (http://web.expasy.org/protparam/, accessed on 4 June 2022). The Cello website (http://cello.life.nctu.edu.tw/cello2go/, accessed on 4 June 2022) was applied to predict the subcellular localization of QmbHLHs.

### 2.2. Phylogenetic Analysis and Multiple Sequence Alignment

Based on the alignments of the amino acid sequences of *Q. mongolica* bHLH proteins by the Clustal W program [32] and MEGA 7.0 software (http://www.megasoftware.net/, accessed on 12 August 2022) [33], the phylogenetic tree was constructed using the neighbor-joining method with 1000 bootstrap repeats and visualized by iTOL (https://itol.embl.de/, accessed on 22 August 2022).

### 2.3. Analysis of Gene Structure and Conserved Motifs

The gene structure display system GSDS (http://gsds.cbi.pku.edu.cn, accessed on 16 July 2022) was used to analyze the structures of the exons and introns of the *QmbHLH* genes. Conserved motif analysis was performed on the extracted bHLH protein sequences using the MEME (http://meme-suite.org/tools/meme, accessed on 16 July 2022); the optimum width of the motifs ranged from 6 to 50, and the maximum number of motifs was set to 10) online tool. NCBI Batch CD-Search (https://www.ncbi.nlm.nih.gov/, accessed on 27 July 2022) was applied to analyze conserved domains. The results were visualized using the program “Gene Structure View (Advanced)” of the TBtools software (Version 1.098769) [34].

### 2.4. Chromosomal Distribution and Synteny Analysis

Chromosomal localizations of QmbHLH genes were determined by using the program Gene Location Visualize from GTF/GFF in TBtools, and the tandemly duplicated gene pairs were linked with a red line. Furthermore, the synteny and collinearity analyses of *Q. mongolica* bHLH family genes were performed using One-step MCScanX [35], and the results were visualized using the Advanced Circos program in TBtools. The non-synonymous rate (Ka), synonymous rate (Ks), and evolutionary rates (Ka/Ks ratio) of the *Q. mongolica* bHLH gene family were determined using KaKs Calculator v2.0 (https://sourceforge.net/projects/kakscalculator2/, accessed on 16 August 2022) [36] to analyze the selection pressure of QmbHLHs. In addition, we downloaded the genome files of A. thaliana (https://www.ncbi.nlm.nih.gov/genome/?term=Arabidopsis+thaliana, accessed on 16 June 2022) and Populus trichocarpa (https://www.ncbi.nlm.nih.gov/genome/?term=Populus%20trichocarpa, accessed on 16 August 2022) from the NCBI website and analyzed the synteny relationship of the bHLH genes between *Q. mongolica* and the two species (*A. thaliana* and *P. trichocarpa*) using the Dual Synteny Plotter program in TBtools.

### 2.5. Analysis of Cis-Elements in Promoters and Prediction of QmbHLH Protein Interaction Network 

The 2000 bp upstream sequences of each putative QmbHLH gene, defined as promoter sequences, were extracted from the genome file of *Q. mongolica* using TBtools. The promoter sequences were submitted to the PlantCARE (http://bioinformatics.psb.ugent.be/web-tools/PlantCARE/html/, accessed on 21 July 2022) website to predict cis-elements. The results obtained were then manually screened for cis-elements and visualized using TBtools. The protein interaction network of QmbHLH proteins was predicted with *A. thaliana* as the comparative organism using STRING (https://string-db.org/, accessed on 13 July 2022).

### 2.6. Tissue-Specific Expression Pattern Analysis

The expression data of QmbHLH genes in different tissues (roots, stems, and leaves) of *Q. mongolica* were obtained from our RNA-seq data (unpublished). The expression abundance of QmbHLH genes were calculated using fragments per kilobase of exon per million fragments mapped (FPKM) for mean values (Table S1). TBtools software was used to standardize the FPKM values and visualize expression data of QmbHLH genes.

### 2.7. Plant Materials and Treatments

*Q. mongolica* seeds were sown in pots filled with soil (nutrient soil: perlite: vermiculite = 3:1:1) and randomly placed in an artificial growth room of Shenyang Agricultural University (41°49′34.90” N; 123°34′18.68” E, Shenyang, China) under cultivation conditions of 16 h/25 °C during the day, 8 h/20 °C at night, 60% relative humidity, and 400 μmol/(m^2^·s) light intensity. After 3 months of growth, the seedlings that were grown consistently were transferred to low temperature (4 °C), drought (20% polyethylene glycol 6000, PEG6000), salt (300 mM NaCl), and weak light (80 μmol/(m^2^·s) light intensity) stress environments. The leaves, stems, and roots were sampled separately after 12, 24, and 48 h of each treatment. Untreated seedlings of *Q. mongolica* at 0 h were used as control plants. Three biological replicates were performed for each experiment. The samples were rinsed with purified water and quickly placed in liquid nitrogen for storage at −80 °C.

### 2.8. RNA Extraction and qRT-PCR Analysis

Total RNA of each sample was extracted using an RNA Isolation Kit (TianGen, Beijing, China). cDNA was synthesized with approximately 1 µg of total RNA in a final volume of 20 μL using PrimeScript^TM^ RT Reagent Kit with gDNA Eraser (TaKaRa, Dalian, China). The qRT-PCR reactions were performed using TB Green Premix Ex TaqTM Ⅱ (TliRNaseH Plus) (TaKaRa, Dalian, China) on a Step One Plus RT-PCR system (Applied Biosystems, Waltham, MA, USA). The reaction program was described by Zhan et al. [37]. The relative expression levels of randomly selected QmbHLH genes were calculated using the 2^−∆∆Ct^ method with the QmUBQ gene as a reference gene [38]. The specific primers of selected genes are shown in Table S2. Three independent replications were performed for each sample.

## 3. Results

### 3.1. Identification and Characterization of QmbHLHs in Q. mongolica

We used BLASTp and hidden Markov model (HMM) analysis to identify *QmbHLH* family genes in *Q. mongolica* and verified the existence of bHLH conserved domains using SMART and NCBI-CDD (Figure S1). These genes were named *QmbHLH01* to *QmbHLH89* in accordance with their positions on the *Q. mongolica* chromosome, and the features of these genes are listed in Table S3. The number of amino acids in the predicted proteins ranged from 91 (QmbHLH12 and QmbHLH67) to 712 (QmbHLH81). The theoretical isoelectric point varied from 4.76 (QmbHLH29) to 10.15 (QmbHLH45). The values of molecular weight ranged from 10253.56 (QmbHLH12) to 79230.63 (QmbHLH81). The instability index ranged from 32.60 (QmbHLH43) to 86.61 (QmbHLH19); therefore, QmbHLH43 was considered the most stable protein. The GRAVY values of nearly all QmbHLH proteins were negative, except for that of QmbHLH32, which was positive, indicating that all QmbHLH proteins except QmbHLH32 were hydrophilic. The analysis of subcellular localization showed that 78 QmbHLHs were located in the nucleus; 2 in mitochondria; 7 in chloroplasts; and 2 in the cytoplasm.

### 3.2. Phylogenetic Analysis, Multiple Sequence Alignment, and DNA-binding Activity

**Figure 1:**
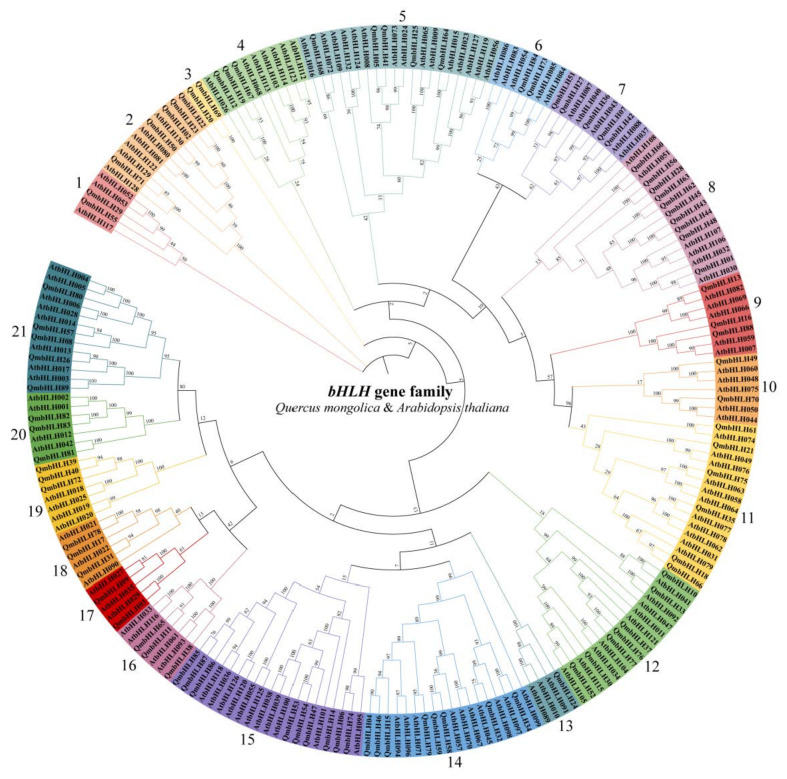
Phylogenetic relationships among the bHLH proteins in *Q. mongolica* and *A. thaliana*. The phylogenetic tree was built in MEGA 7.0 via the N-J approach with 1000 bootstrap replicates. All QmbHLH genes were grouped into 21 subgroups according to the topological structure of the tree and classification rules of the Arabidopsis *bHLH* genes.

To further investigate the evolutionary associations of the *Q*. *mongolica* bHLH transcription factor family, a phylogenetic tree was constructed in accordance with the protein sequences of 127 AtbHLHs and 89 QmbHLHs, using the N-J method (Figure 1). The bHLH proteins were clustered into 21 subgroups according to tree topology, together with the classification of bHLHs in *A*. *thaliana* [7]. Among the 21 subgroups, subgroup 13 had the lowest number of members (1 QmbHLH), while 8 had the highest number of members (10 QmbHLHs). None of the AtbHLHs were classified in subgroup 3, which consisted of two QmbHLHs (namely, QmbHLH20 and QmbHLH69). This suggests a novel evolutionary direction for *Q*. *mongolica*.

**Figure 2:**
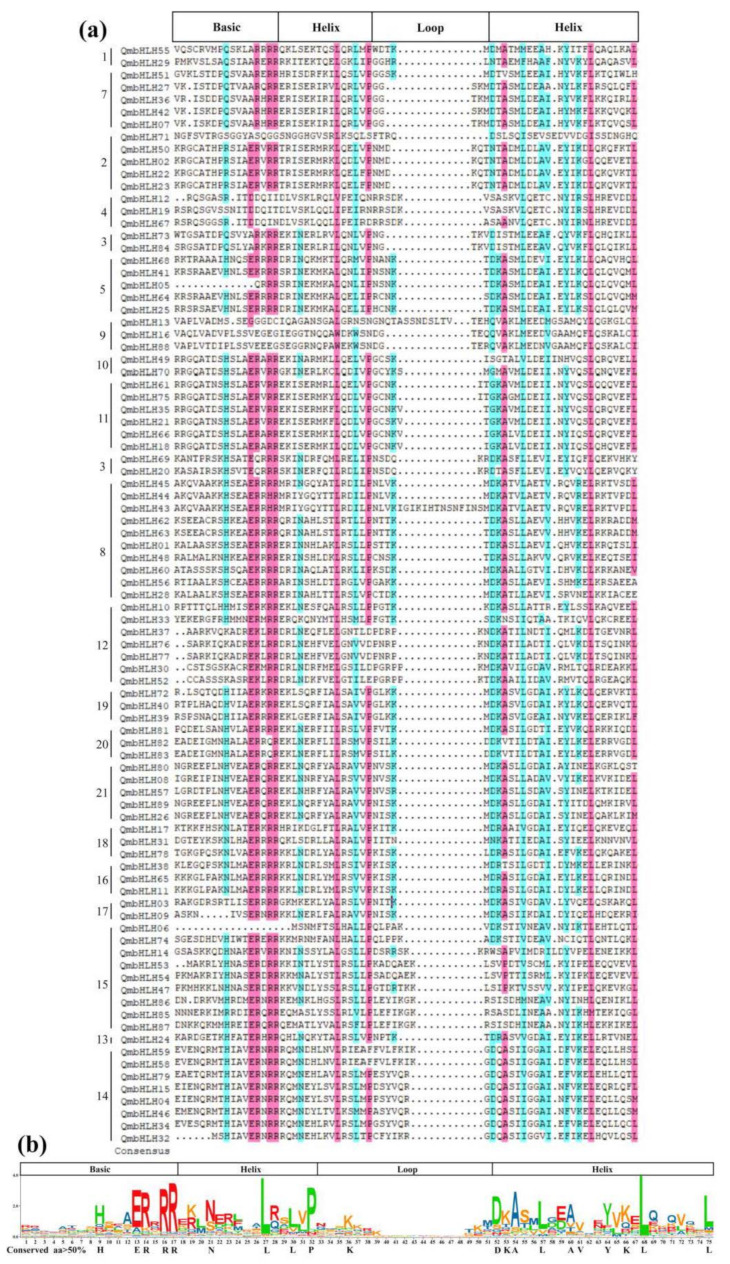
Multiple sequence alignment of amino acid sequences of the conserved structural domains of QmbHLH proteins from *Q. mongolica*. (**a**) Multiple sequence alignment of QmbHLH proteins. The numbers 1–21 in the first column from the left in (**a**) indicates the QmbHLH proteins were classified into 21 groups based on the clustering results of Figure 1. A purple background indicates a consensus ratio of >75%, while a light blue indicates a consensus ratio of >50%. (**b**) Sequence identifier of the QmbHLH structure field. The total height of each stack denotes the preservation of the sequence at that location. The uppercase letters in (**b**) indicates the frequency of individual bases. The bold letters below represent a consensus ratio for the conserved sites in the bHLH structural domain of >50%.

Multiple sequence alignment of the QmbHLH protein structural domain indicated that the structural domain was composed of four conserved regions, namely two helical regions, a loop region, and a basic region (Figure 2a). Furthermore, the consensus ratio of the 20 amino acid residues conserved in the bHLH structural domain was greater than 50% (Figure 2b). Among these residues, Glu-13, Arg-17, Arg-16, Arg-14, Pro-32, Ala-54, Leu-70, Leu-77, and Leu-27 were highly conserved, with a consensus ratio greater than 75%. Among the 20 conserved amino acid residues, three (Asn-21, Pro-32, and Leu-27) were identified in the first helix; five (Glu-13, His-9, Arg-17, Arg-16, and Arg-14) in the basic region; one (Lys-36) in the circular region; and ten (Asp-52, Lys-53, Leu-68, Ala-54, Ala-60, Val-61, Leu-57, Leu-75, Lys-66, and Tyr-64) in the second helix.

In accordance with the *A*. *thaliana* classification criteria, the DNA-binding activity of the basic *QmbHLH* region was examined [7]. According to the presence of residues in the basic region of the bHLH structural domain, 71 and 10 QmbHLHs were categorized as E-box-binding and non-E-box-binding proteins, respectively (Table S3). Furthermore, the 62 E-box-binding proteins were divided into 53 G-box-binding and 18 non-G-box-binding proteins. Additionally, 8 of the 89 QmbHLHs were categorized as non-DNA-binding proteins.

### 3.3. Analysis of QmbHLH Gene Structure and Conserved Motifs 

**Figure 3:**
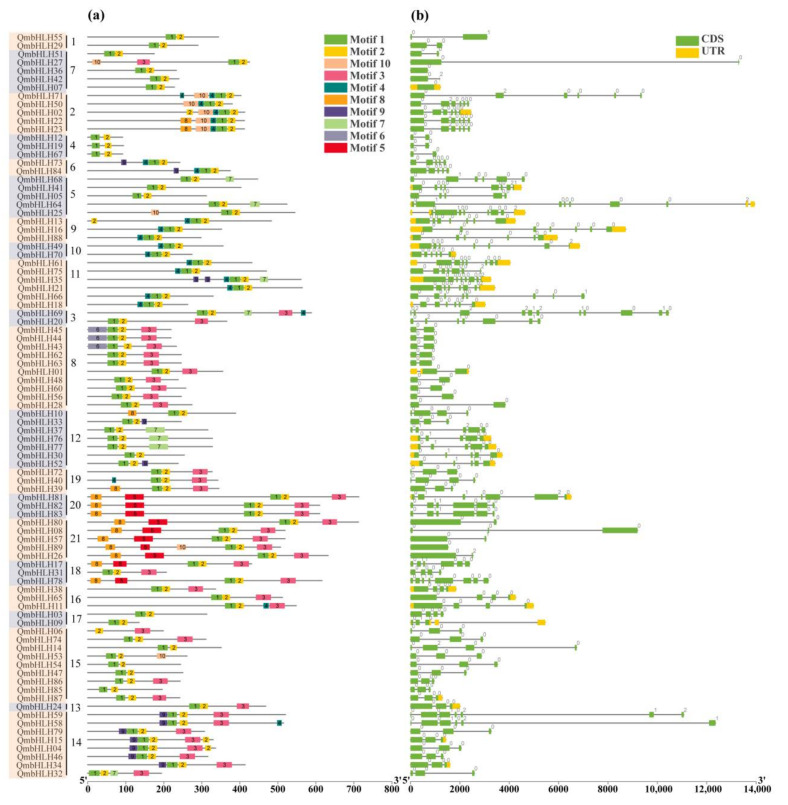
Conserved patterns and architectures of *QmbHLH* genes. (**a**) Conserved patterns in the QmbHLH proteins. The numbers 1–21 in the second column from the left in (**a**) indicates the *QmbHLH* genes were classified into 21 groups based on the clustering results of Figure 1. These motifs, numbered 1–10, are indicated with various colored boxes. The sequence identity of each pattern is exhibited in Figure S2. (**b**) Exon–intron architecture of *QmbHLH* genes. UTRs, introns, and exons are indicated with yellow rectangles, lines, and green rectangles, respectively; the numbers (0–2) represent the intron phase.

Ten motifs of the QmbHLH proteins were predicted, using the online program MEME (Figure 3a). Almost all QmbHLH proteins contained motifs 1 and 2, except for QmbHLH06, and the motifs were very close to each other, which might have an important effect on QmbHLH proteins. The various subgroups exhibited distinctive patterns. For instance, the QmbHLH proteins in subgroup 20 exhibited patterns 1, 2, 3, 5, and 8. Some patterns were unique and appeared only in individual subgroups. Pattern 6 was present only in subgroup 8; therefore, this pattern may perform a specific function. The existence of similar patterns in the same subgroup indicates that the corresponding QmbHLHs have similar functions. Conversely, some small differences between QmbHLHs in the same subgroup were revealed. For example, QmbHLH13 exhibited an extra pattern 2, in addition to the common patterns 1, 2, and 4 in subgroup 9. QmbHLH89 exhibited pattern 10, in addition to the common patterns 1, 2, 3, 5, and 8 in subgroup 21.

To understand the architectural composition of *QmbHLH*, we examined the exon patterns derived by comparing genomic sequences (Figure 3b). The number of exons varied from 1 to 11. Only four *QmbHLH* genes had one exon, while the remaining had two or more. *QmbHLH* genes with one intron (*n* = 20) and two introns (*n* = 20) comprised the highest proportion of all *QmbHLH* genes. The *QmbHLH69* gene contained 10 introns, the most of all the *QmbHLH* genes. The three intronless genes (*QmbHLH07/36/89*) belonged to two subgroups 7 and 21. The number of exons and introns of the *QmbHLH* genes within subgroups 1, 2, 4, and 16 was consistent. 

### 3.4. Chromosomal Distribution and Synteny Analysis 

**Figure 4:**
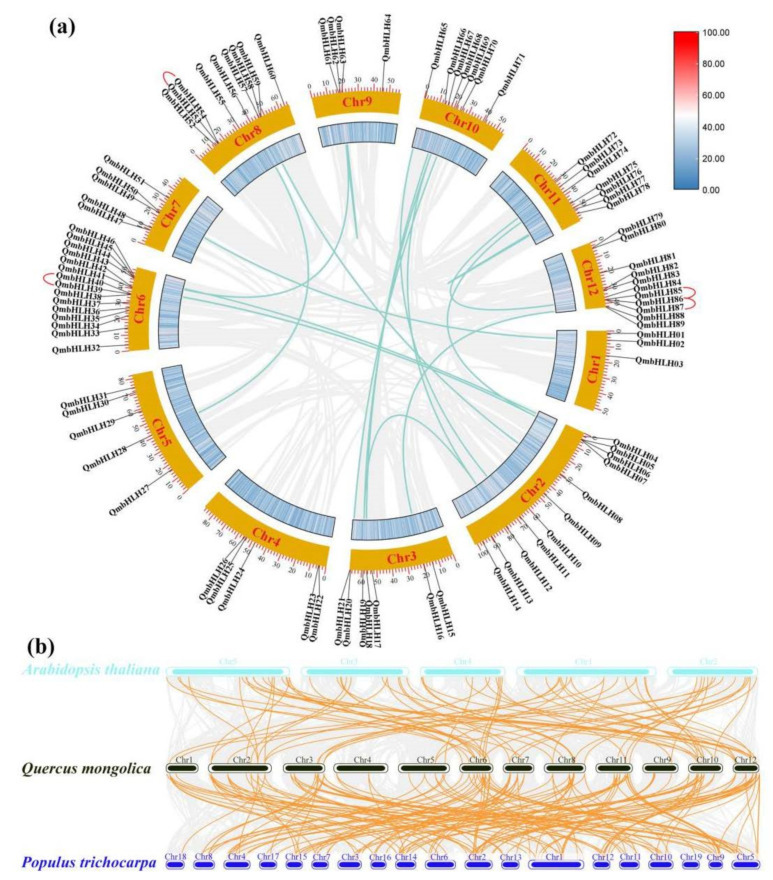
(**a**) Interchromosomal correlations and chromosomal distribution of the *QmbHLH* genes. Tandem duplicated genes are indicated with a red line. Gray lines represent all the synteny blocks in the genome of *Q. mongolica*, while turquoise lines indicate the segmentally duplicated *QmbHLH* gene pairs. Heat maps represent the density of genes on chromosomes. The yellow circles represent chromosomes. The number of chromosomes is shown in the middle of each chromosome. The scale on the circle is in terms of MegaBase (Mb) and represents chromosome length. (**b**) The *bHLH* gene synteny analysis of *P. trichocarpa*, *A. thaliana*, and *Q. mongolica*. In the background, the gray lines represent the collinear blocks between the genomes of *P. trichocarpa*, *A. thaliana*, and *Q. mongolica*. The collinear *bHLH* gene pairs are highlighted with orange lines. The chromosome numbers are on the left side of each chromosome.

All *QmbHLH* genes were heterogeneously distributed across the 12 *Q*. *mongolica* chromosomes (Figure 4a). Fifteen *QmbHLH* genes (the largest number) appeared on Chr6, whereas only three *QmbHLH* genes were located on Chr1. The genes positioned on the other chromosomes varied from 4 to 11 in number. Gene duplication events, including tandem duplication, genome duplication, transposon duplication, and fragment duplication, are universal events that occur in all organisms during evolution [39,40]. Replication plays a critical role in the expansion of *QmbHLHs*. Among the 95 *QmbHLH* genes, we acquired 4 tandem replicator pairs (Figure 4a and Table S4) and 18 fragment replicator pairs (Figure 4a and Table S5). To estimate the selection pressure of the *QmbHLH* genes during evolution, we calculated the ratio of Ka (non-synonymous) to Ks (synonymous) for 18 *QmbHLH* homologous gene pairs (Table S5). The Ka/Ks values for the *QmbHLH* gene pairs were usually <1, which indicates that purifying selection occurred during evolution.

To further elucidate the origin and evolutionary mechanism of the QmbHLH family, synteny analysis of *Q. mongolica* and two other species (*A*. *thaliana* and *P*. *trichocarpa*) was performed (Figure 4b). Based on sequency homology, 75 and 167 parallel gene pairs generated from 48 and 75 QmbHLH genes exhibited synthetic associations with the genes of *A*. *thaliana* (Table S6) and *P*. *trichocarpa* (Table S7), respectively. In addition, some collinear pairs formed by the 44 *QmbHLH* genes were detected between *Q. mongolica* and both *A*. *thaliana* and *P*. *trichocarpa*, indicating that these orthologous pairs may have existed prior to ancestral differentiation. Some *QmbHLH* genes (*QmbHLH9/14/24/25/40/44/45/53/54/68/86/87*) exhibited no collinearity with genes in *A*. *thaliana* or *P*. *trichocarpa*, suggesting that they may be unique to the evolution of *Q. mongolica* compared to *A*. *thaliana* and *P*. *trichocarpa*.

### 3.5. Analysis of Cis-Elements and Prediction of Protein Interaction Network

**Figure 5:**
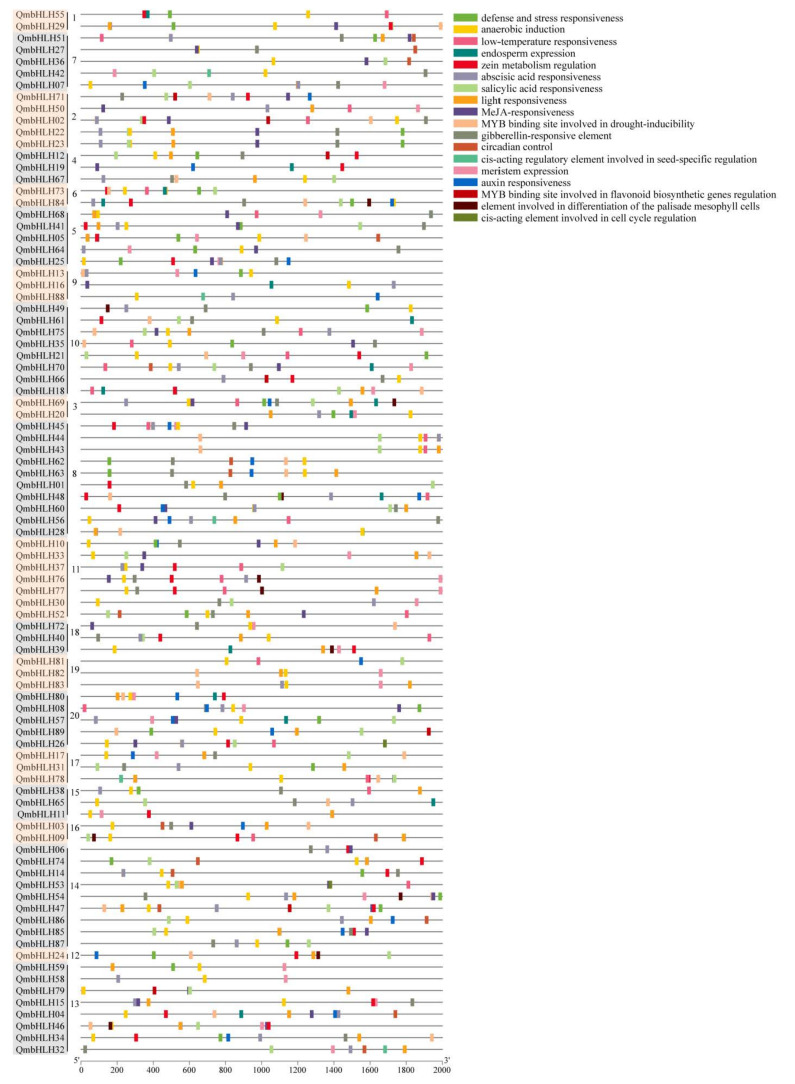
Cis-acting elements in the promoter region 2000 bp upstream of *QmbHLH* genes. Various colored rectangles denote distinct cis-acting elements; some elements are overlapping. The numbers 1–21 in the second column from the left indicates the *QmbHLH* genes were classified into 21 groups based on the clustering results of Figure 1.

To predict the functions of *QmbHLH* genes, putative cis-elements were analyzed and identified in the promoter region in the 2000 bp range upstream of the *QmbHLH* genes using PlantCARE (Figure 5). The cis-acting elements were classified into three groups, namely five phytohormone-associated elements, eight plant-growth- and development-related elements, and five stress-response-associated elements. Among them, the plant growth and development-associated group and phytohormone-associated group had the most cis-acting elements. The stress-response-related group contained abscisic-acid-responsive elements, found in 75 *QmbHLH* genes; light-responsive elements, found in 76 *QmbHLH* genes; and elements essential for anaerobic induction, found in 82 *QmbHLH* genes.

**Figure 6:**
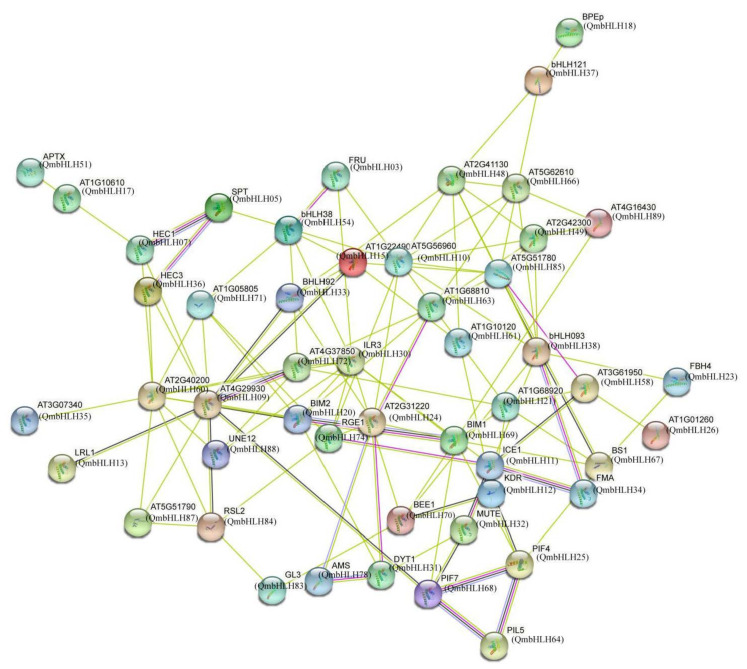
Protein interaction network based on orthologs of QmbHLHs in *Arabidopsis*. The network nodes represent proteins. The color of the lines between network nodes indicates the source of protein interaction evidence. Cyan line presents data from curated databases, purple line presents data from experimentally determined, green line gene neighborhood, red line gene fusions, blue line gene co-occurrence; yellow line presents text mining, black line co-expression, and gray line protein homology.

To better understand the functions of QmbHLHs, an interaction network of 89 QmHLHs, based on homologous proteins of *A*. *thaliana*, was predicted using STRING (Figure 6). QmbHLH68, QmbHLH64, and QmbHLH25 were homologous to phytochrome-interacting factor 7 (PIF7), PIF3-like 5 (PIL5), and phytochrome-interacting factor 4 (PIF4), respectively. PIF7, PIF3, and PIL5 are key transcription factors involved in light and phytohormone signaling pathways. By interacting with QmbHLH25, QmbHLH64 and QmbHLH68 might regulate QmbHLH12. QmbHLH12 was homologous to KDR, a critical regulator of light signaling and shade avoidance, and inhibits the activity of the bHLH transcription factor HFR1. QmbHLH11 was homologous to ICE1. QmbHLH34 and QmbHLH32 were homologous to FMA and MUTE, respectively, which were key proteins for regulating stomatal formation. To respond to cold stress, QmbHLH11 may regulate stomatal formation by interacting with QmbHLH34 and QmbHLH32. In general, the interaction network provides an important reference for studying the regulatory mechanisms of QmbHLHs.

### 3.6. Patterns of QmbHLH Expression in Different Tissues

**Figure 7:**
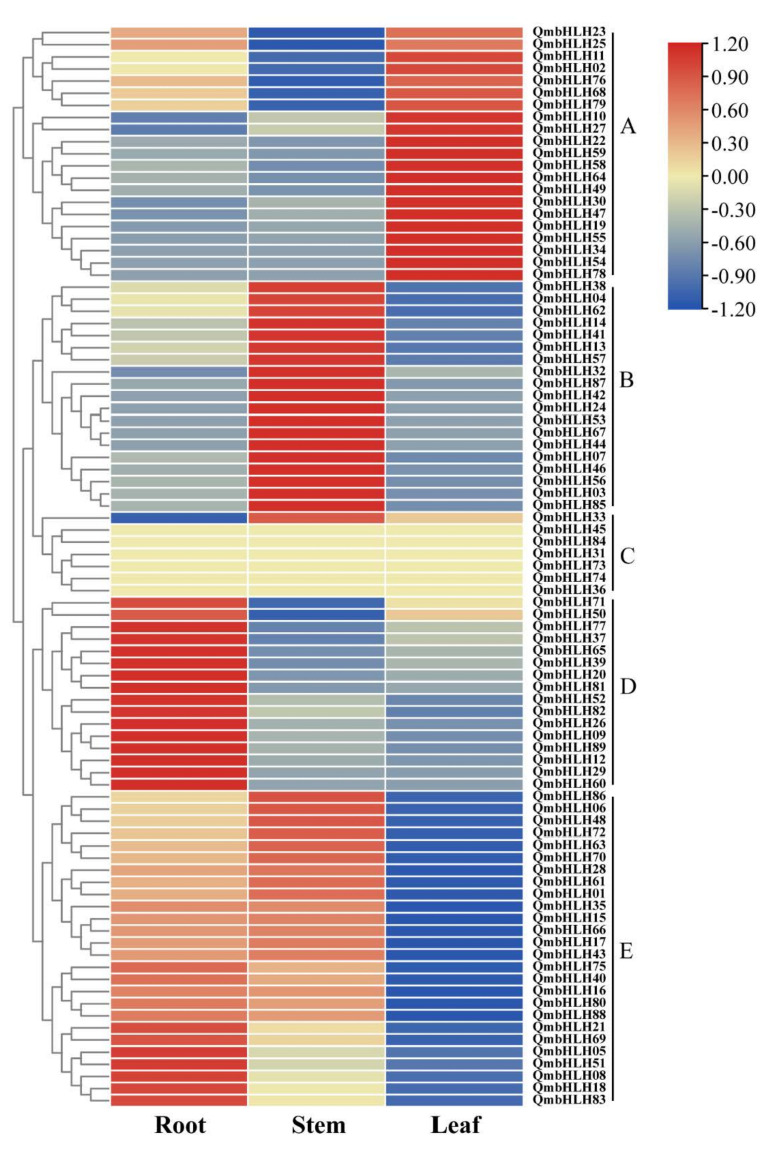
Expression profiles of *QmbHLH* genes in various tissues. The heat map was constructed according to the standardized FPKM values with clustering. The transition from blue to red represents diverse levels of expression. *QmbHLH* expression patterns were classified into five major groups and represented by A-E based on cluster analysis.

To better understand the underlying functions of *QmbHLHs*, we examined the levels of *QmbHLH* gene expression using unpublished transcriptomic data from root, stem, and leaf tissues of *Q*. *mongolica* (Figure 7 and Table S1). *QmbHLH* expression patterns were classified into five major groups based on cluster analysis. 

Almost all *QmbHLH* genes were identified in the root, stem, or leaf tissues, except for the six *QmbHLH* genes (*QmbHLH45/84/31/73/74/36*) in group C. The *QmbHLH* genes in group A were clustered together since they exhibited high expression levels in leaf tissues. In contrast, the *QmbHLH* genes in group E were clustered together since they exhibited low expression levels in leaf tissues. The *QmbHLH* genes in group B were clustered together since they exhibited high expression levels in stem tissues. In addition, the *QmbHLH* genes in group D were clustered together since they exhibited high expression levels in root tissues. Expression analysis suggested that different subgroups of *QmbHLH* genes may exert various effects on the growth, progression, and stress response of *Q*. *mongolica*.

### 3.7. Expression of QmbHLHs in Response to Different Abiotic Stresses 

**Figure 8:**
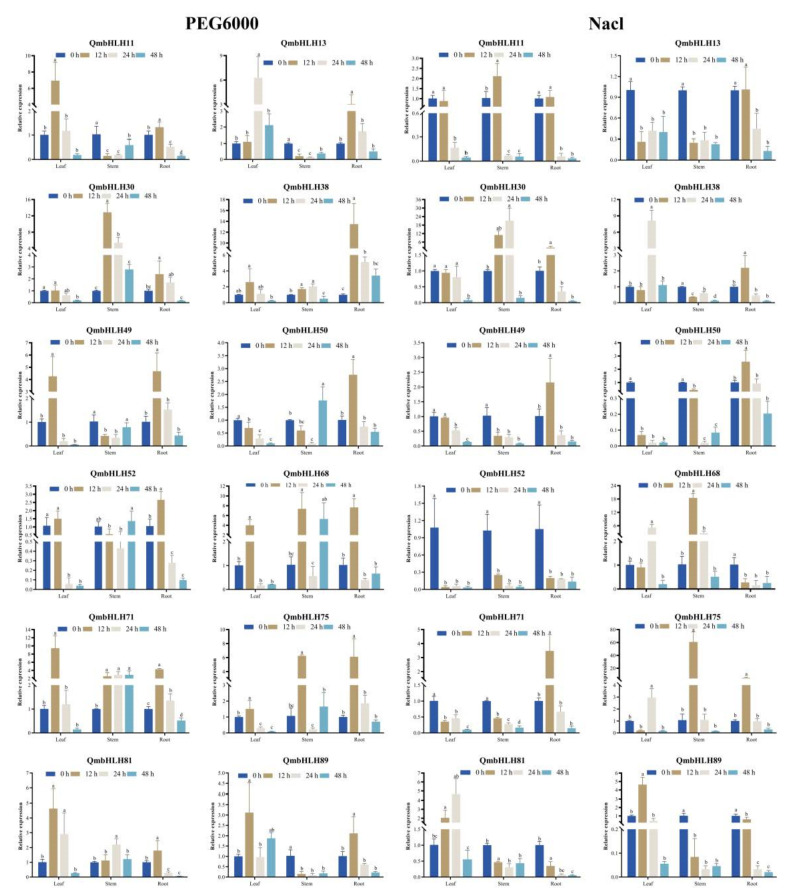
Expression patterns of 12 *QmbHLH* genes in leaf, stem, and root tissues under PEG6000 and NaCl treatments via qRT-PCR. Different letters in each treatment indicate significant differences in *QmbHLH* gene expression (*p* < 0.05, Duncan’s test).

**Figure 9:**
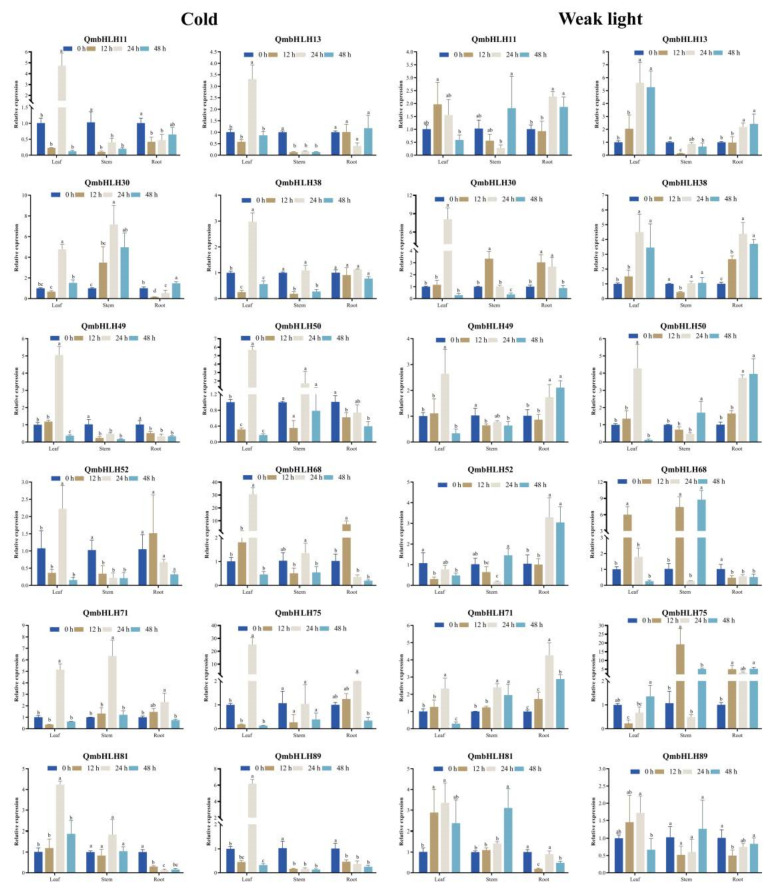
Expression patterns of 12 *QmbHLH* genes in leaf, stem, and root tissues under cold and weak light treatments via qRT-PCR. Different letters in each treatment indicate significant differences in *QmbHLH* gene expression (*p* < 0.05, Duncan’s test).

To determine whether *QmbHLH* gene expression was influenced by various abiotic stresses, we examined the expression patterns of 12 *QmbHLH* genes in stem, leaf, and root tissues in response to PEG6000, NaCl, cold, and weak light treatments, using qRT-PCR (Figure 8 and Figure 9). 

The expression of most *QmbHLH* genes was remarkably inhibited or induced by the various treatments and varied during the early phases of treatment. Some *QmbHLH* expression patterns changed over time or appeared in different organs in response to the different stresses. For example, in response to PEG6000 treatment, the expression of *QmbHLH81* and *QmbHLH30* exhibited a marked increase, followed by a subsequent decrease. *QmbHLH11* expression markedly increased and subsequently decreased in the leaf and root tissues; conversely, *QmbHLH11* expression significantly decreased, then increased in stem tissues. Most *QmbHLH* genes were upregulated in leaf, stem, and root tissues under different stress treatments; only *QmbHLH13* and *QmbHLH52* were downregulated in all tissues in response to NaCl treatment. Interestingly, some genes exhibited opposite expression patterns in response to various treatments. For example, the expression of *QmbHLH89* first increased, then decreased, and finally increased again in PEG-treated leaves. However, the expression of *QmbHLH89* decreased, increased, and then decreased in cold-treated leaves. In addition, *QmbHLH89* expression was upregulated and then downregulated in NaCl-treated leaves. The expression of other genes was highly variable in specific organs. For example, *QmbHLH68* and *QmbHLH75* expression in leaves and stems responded significantly to NaCl treatment. Furthermore, correlations between *QmbHLH* gene expression patterns were examined (Figure S3). Most *QmbHLH* genes were positively correlated, except for a few that were negatively correlated. There was a remarkable positive association between some *QmbHLH* genes, such as *QmbHLH49* and *QmbHLH38*/*QmbHLH11*/*QmbHLH89*/*QmbHLH52*/*QmbHLH71*/*QmbHLH50* (*p* < 0.05).

## 4. Discussion

The family of bHLH transcription factors is the second-most extensive family in eukaryotes [10,41]. Previous studies on *bHLH* gene families have determined the number of *bHLH* genes in various plants, including *A*. *thaliana* (162) [42], *Cucumis mleo* (118) [43], *Helianthus annuus* (183) [44], *Ficus carica* (118) [41], *S*. *bicolor* (174) [20], *Cucumis sativus* (142) [45], *Ginkgo biloba* (85) [46], *Carthamus tinctorius* (41) [47], and *Prunus mume* (95) [48]. bHLH transcription factors are extensively involved in several biological processes, such as organ development, hormone synthesis, light signaling, and defense against abiotic and biotic stresses. However, no functional determination studies of the *bHLH* gene family in *Q*. *mongolica* have been reported. In the current study, we identified 89 *QmbHLH* genes, providing a better understanding of the function of the *bHLH* gene family in *Q*. *mongolica*.

According to the phylogenetic analysis of *A*. *thaliana* bHLH proteins and the topological structure of the phylogenetic tree, 89 proteins were clustered into 21 subgroups (Figure 1). We observed at least one QmbHLH protein in each *AtbHLH* subgroup, indicating that the bHLH family may have differentiated earlier than *A*. *thaliana* and *Q*. *mongolica* diverged. However, not all QmbHLH proteins (QmbHLH69 and QmbHLH20) were included in the subgroups containing AtbHLHs, indicating that *A*. *thaliana* and *Q*. *mongolica* differed in terms of their evolutionary processes. Nearly all members of the QmbHLH family include motifs 1 and 2 (Figure 3a). This is consistent with results obtained from tomato, maize, barley, and ginkgo [46,49,50,51], suggesting that QmbHLH transcription factors are evolutionarily conserved in plants. 

Exon–intron structural analysis of genes can provide important insights into phylogenetic relationships [46], and members of the same subfamily possess similar gene architecture [52]. Diversification of exon–intron structures plays an essential role in gene family evolution and consists of three main pathways, namely insertion/deletion, exonization/pseudoexonization, and exon gain/loss [53]. The exon–intron number of *Oryza sativa bHLH* genes varies from 1 to 4 [54], while that of apple *bHLH* genes varies from 0 to 19 [55]. In the present study, the exon–intron number of the *QmbHLH* genes varied from 0 to 11 (Figure 3b), indicating that the exon–intron structures of *QmbHLH* genes experienced insertion/deletion or exon gain/loss during evolution.

DNA-binding activity is dictated by the basic region of the bHLH structural domain, while the HLH region is involved in the generation of homodimers or heterodimers [4]. Previous studies [6,7] have classified QmbHLHs into either non-E-box binding or E-box-binding, based on the presence of two critical amino acid residues, Arg-16 and Glu-13, in the basic region (Figure 2a). If the basic region consists of only one of these residues, the bHLH is categorized as a non-E-box-binding protein. In addition, E-box-binding proteins can be further classified as G-box binding or non-G-box binding based on the presence of Arg-17, Glu-13, and His/Lys-9 residues or the absence of His/Lys-9 residues, respectively [1,7]. Furthermore, QmbHLHs are classified as non-DNA-binding proteins if the basic domain contains less than four basic amino acids and includes neither or only one of Glu-13 and Arg-16. In this study, 53 QmbHLHs (59.55%) were categorized as G-box-binding proteins; 18 (20.22%) as non-G-box-binding proteins; 10 (11.23%) as non-E-box-binding proteins; and the remaining 8 (8.98%) as non-DNA-binding proteins (Table S3). G-box-binding proteins comprised the largest proportion of the QmbHLHs, which is comparable to the results for *O*. *sativa* and *A*. *thaliana* [54]. Previous studies have revealed that residues Leu-61 and Leu-27 in the helix region play an essential role in the generation of heterodimers or homodimers among bHLHs or between bHLHs and other transcription factors [7,56]. These residues are the most conserved residues among bHLHs in plants [4,5]. Among the QmbHLHs, residues Leu-68 and Leu-27 were 97.7% and 96.6% conserved, respectively. These residues were less conserved than those of *P*. *mume* (98% and 97%, respectively) [48] and *Citrus reticulata* (100% and 100%, respectively) [56]. This implies that QmbHLHs possess dimerization ability.

Gene duplication events are essential for gene family expansion and genome evolution during plant evolution [57]. Tandem duplication and segmental replication were the main replication modes observed in this study. In the *QmbHLH* family, we identified 4 tandem duplication gene pairs (Figure 4a and Table S4) and 18 segmental duplication gene pairs (Figure 4a and Table S5). These results indicate that segmental duplication may be a more important driver of *QmbHLH* gene family expansion. There were 167 collinear gene pairs among the *bHLH* genes of *Q*. *mongolica* and *P*. *trichocarpa* and 75 collinear gene pairs among the *bHLH* genes of *Q*. *mongolica* and *A*. *thaliana*, indicating that *Q*. *mongolica* had a more extensive synteny relationship with *P*. *trichocarpa* than with *A*. *thaliana* (Figure 4b). Typically, the gene function of a clade is highly, but not completely, conserved across plant species [45]. Consequently, it is essential to accurately recognize genes that are homologous across plant species via comprehensive analysis.

Gene expression profiling can be used for functional analysis [20,58]. Thus, we used transcriptome data from different tissues (roots, stems, and leaves) of *Q*. *mongolica* to analyze *QmbHLH* gene family expression. The expression pattern of each *QmbHLH* gene exhibited a clear tissue specificity (Figure 7). For instance, *QmbHLH32* and *QmbHLH34* were highly expressed in stems and leaves, respectively. The homologous genes *MUTE* and *FMA* control the differentiation of meristematic tissues during stomatal development [59,60]. In addition, *QmbHLH83* was highly expressed in the roots. The homologous gene *GL3* promotes the generation of non-hairy root epidermal cells along with *MYB66/WER* and the generation of hairy root epidermal cells along with *CPC* [61]. These results further expand our understanding of the functions of *QmbHLH* genes.

Abiotic stress is the main factor limiting plant growth and progression [62]. The bHLH transcription factor family has numerous members with diverse functions. In two model plants, *O*. *sativa* and *A*. *thaliana*, the responses of bHLH to abiotic stresses has received extensive attention. *AtbHLH122* is induced by various stresses, such as osmosis, drought, and high salt stress. Transgenic plants overexpressing *AtbHLH122* have higher salt and osmotic resistance than wild-type plants [63]. Under drought conditions, *OsbHLH148* expression is increased, which regulates the expression of genes associated with the jasmonate signaling pathway and increases the degree of tolerance of the plant [16]. The roles of bHLH transcription factors in other species require further investigation. In the current study, we selected 12 *QmbHLH* genes and examined their response to four abiotic stresses in various organs. The results showed that the expression levels of nearly all *QmbHLH* genes differed dramatically in different tissues. For example, in response to drought stress, 9 *QmbHLH* genes exhibited elevated expression in leaves, 6 in stems, and 12 in roots. Analysis of the expression patterns revealed the existence of a complicated cross-regulatory network of *QmbHLH* genes. *QmbHLH75* and *QmbHLH68* responded significantly to PEG, NaCl, cold, and low-light treatments, indicating that they may play important synergistic or antagonistic regulatory roles under a variety of stress conditions. Intriguingly, most *bHLH* genes in *S*. *bicolor* exhibit significant downregulation under various stresses [20], contrary to our results, suggesting that *bHLH* genes may form regulatory networks that have different mechanisms in different species. However, these regulatory mechanisms and functions require further in-depth analysis. This part of the study has some limitations. The expression pattern and function of the same gene may differ at different stages of plant growth [64]. In this study, the expression patterns of 12 *QmbHLH* genes were analyzed in very young plants (3 months old). *Q*. *mongolica* is a perennial deciduous woody plant, and these *QmbHLH* genes should also be verified in adult *Q*. *mongolica* plants.

## 5. Conclusions

In the present study, we conducted the first systematic and comprehensive genome-wide analysis of the bHLH gene family in *Q*. *mongolica*. Eighty-nine *QmbHLH* genes were characterized and categorized into 21 subgroups. *QmbHLHs* within the same subgroup had similar gene structures and conserved patterns, which supports the reliability of classification based on phylogenetic analysis. The 89 *QmbHLH* genes were unevenly distributed across the 12 *Q*. *mongolica* chromosomes. Some *QmbHLH* genes might be involved in gene replication events; segmental replication was more favorable than tandem replication for *QmbHLH* expansion. Analysis of protein interactions and synthetic associations further clarified the basic evolutionary mechanisms and functions of QmbHLHs. In addition, tissue-specific gene expression profiles indicated that *QmbHLH* genes may be broadly involved in tissue development. Finally, qRT-PCR results showed that 12 *QmbHLH* genes were actively involved in the abiotic stress response mechanism of *Q*. *mongolica*. This finding provides novel insights into *Q*. *mongolica* under abiotic stress. The results of this study provide a basis for understanding the origin and evolution of *QmbHLH* genes in *Q*. *mongolica* and will facilitate future research on the function of QmbHLHs.

## Data Availability

Genome database have been deposited in the National Center for Biotechnology Information (NCBI) with the BioProject number of PRJNA609556. Other datasets analyzed during the current study are available from the corresponding author on reasonable request.

## Supplementary Materials

The following supporting information can be downloaded at: https://www.mdpi.com/article/10.3390/cimb45020075/s1, Figure S1: Conserved domain analysis of QmbHLHs; Figure S2: Sequence logos of 10 conserved motifs detected in the QmbHLHs; Figure S3: The correlations between *QmbHLH* gene expression patterns; Table S1: FPKM values of *QmbHLH* genes in different tissues; Table S2: Primers used in the qRT-PCR; Table S3: Characteristic information of identified *QmbHLH* genes in Mongolian oak; Table S4: The tandem duplication events of *QmbHLH* genes; Table S5: The 18 pairs of segmental duplicates in *QmbHLH* genes; Table S6: One-to-one orthologous relationships between *Quercus mongolica* and *Arabidopsis thaliana*; Table S7: One-to-one orthologous relationships between *Quercus mongolica* and *Populus trichocarpa*.
