# Supplementary figures and images for: Genome-Wide Identification and Expression Analysis of the bHLH Transcription Factor Family and Its Response to Abiotic Stress in Mongolian Oak (Quercus mongolica)
Source: Curr Issues Mol Biol. 2023 Jan 31;45(2):1127–48. doi: 10.3390/cimb45020075 (PMC9955707; doi:10.3390/cimb45020075)

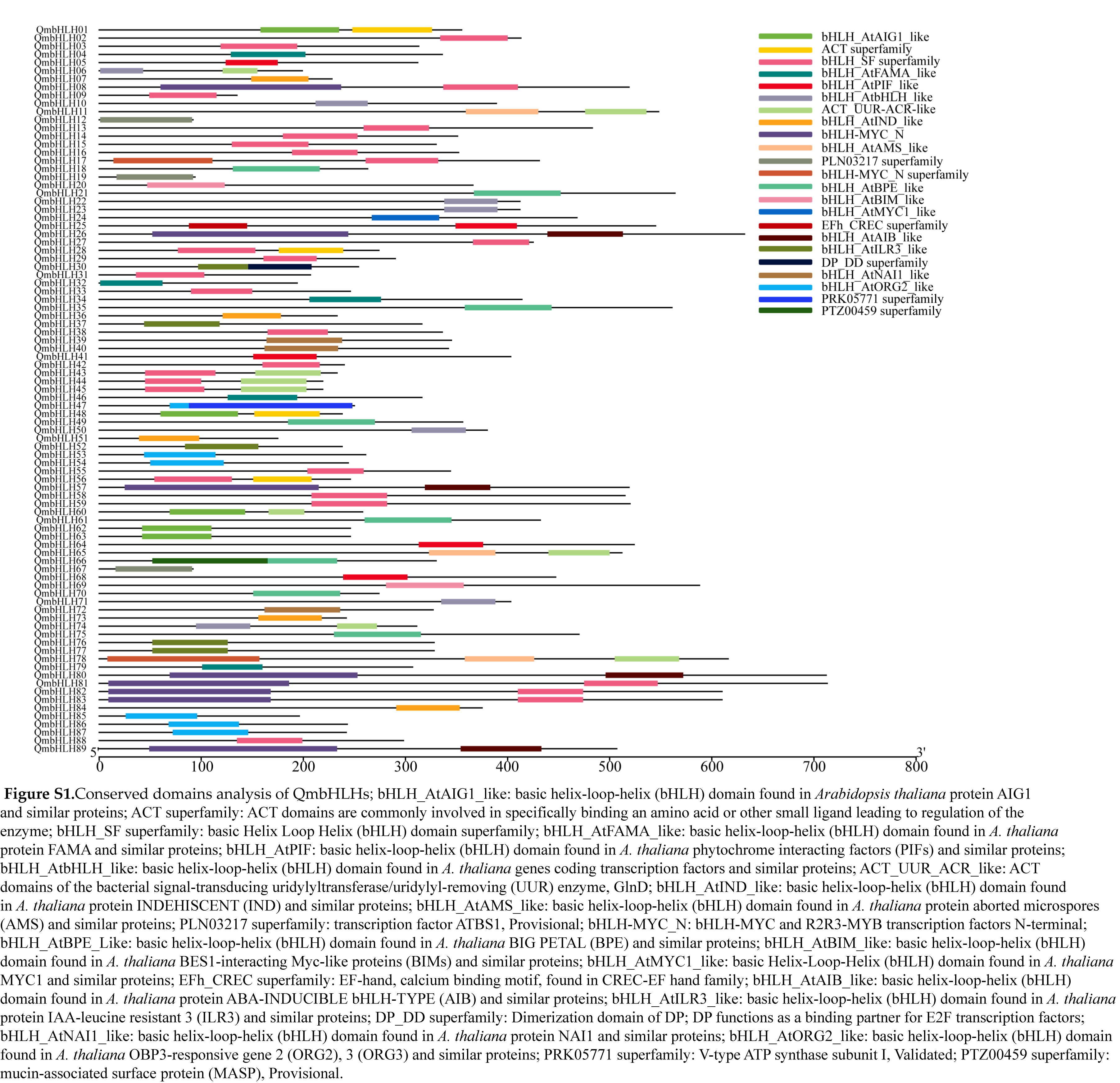

Supplement: Supplementary file 1 [file cimb-45-00075-s001.zip › Figure S1.tif]

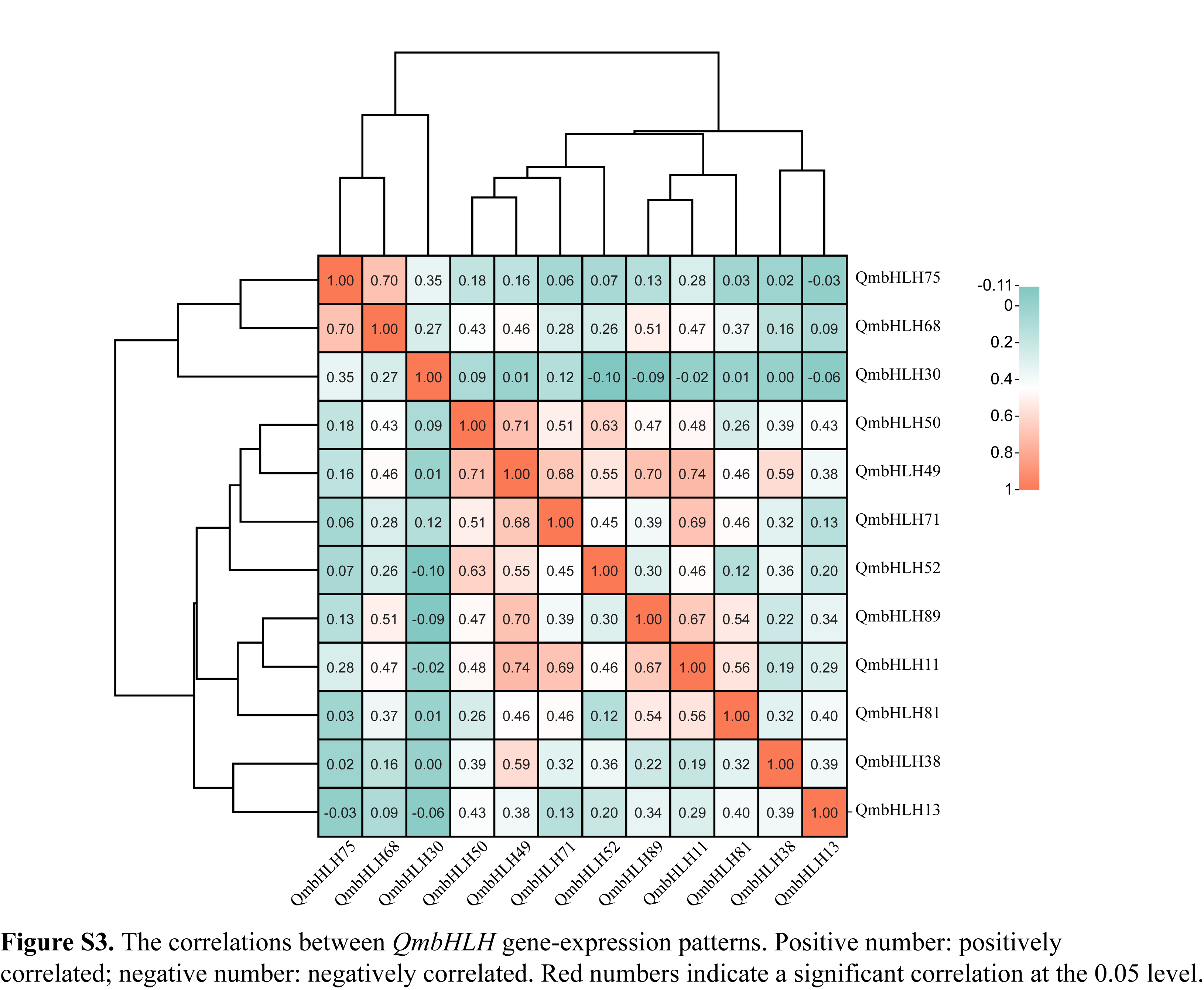

Supplement: Supplementary file 1 [file cimb-45-00075-s001.zip › Figure S3.tif]
